# Discovery of a Novel CCR5 Antagonist Lead Compound Through Fragment Assembly

**DOI:** 10.3390/molecules13102426

**Published:** 2008-10-01

**Authors:** Yanqing Liu, Enkun Zhou, Kunqian Yu, Jin Zhu, Yu Zhang, Xin Xie, Jian Li, Hualiang Jiang

**Affiliations:** 1 School of Pharmacy, East China University of Science and Technology, 130 Mei Long Road, Shanghai 200237, P.R. China; E-mails: yanqing_liu@163.com (Y. L.), jinzh@mail.ecust.edu.cn (J. Z.), yuloveyou8133@sina.com (Y. Z.); 2 The National Center for Drug Screening, Chinese Academy of Sciences, Shanghai 201203, P.R. China; E-mail: nickey838@sina.com (E. Z.); 3 School of Biotechnology, Southwest University, Chongqing 400715, P.R. China; 4 Drug Discovery and Design Center, Shanghai Institute of Materia Medica, Chinese Academy of Sciences, 555 Zu Chong Zhi Road, Shanghai 201203, P.R. China; E-mail: kqyu@mail.shcnc.ac.cn (K-Q. Y.), hljiang@mail.shcnc.ac.cn (H-L. J.)

**Keywords:** CCR5 antagonist, fragment assembly, HIV-1, molecular modeling

## Abstract

CCR5, as the major co-receptor for HIV-1 entry, is an attractive novel target for the pharmaceutical industry in the HIV-1 therapeutic area. In this study, based on the structures of maraviroc and 1,4-bis(4-(7-chloroquinolin-4-yl)piperazin-1-yl)butane-1,4- dione (**1**), which was identified using structure-based virtual screening in conjunction with a calcium mobilization assay, a series of novel small molecule CCR5 antagonists have been designed and synthesized through fragment assembly. Preliminary SARs were obtained, which are in good agreement with the molecular binding model and should prove helpful for future antagonist design. The novel scaffold presented here might also be useful in the development of maraviroc-derived second generation CCR5 antagonists.

## Introduction

Almost a decade ago now, the chemokine receptor CCR5 was identified as the major co-receptor for HIV-1 entry, besides the cellular CD4 receptor [1,2,3,4,5]. CCR5 plays an integral role in the R5-tropic HIV-1 entry process by serving as a critical co-receptor for the viral envelope protein gp120 [6,7]. The natural ligands of CCR5 (RANTES, MIP-1α, MIP-1β) [8] and their derivatives [9,10], as well as some specific monoclonal antibodies against certain epitopes of CCR5 [11] possess anti-HIV-1 activity, and homozygous individuals with a 32-base pair deletion in the gene encoding CCR5 do not express the functional receptor and are ultimately resistant to R5-tropic HIV-1 infection [12]. These facts have made CCR5 an attractive novel target for the pharmaceutical industry in the HIV-1 therapeutic area.

**Figure 1:**
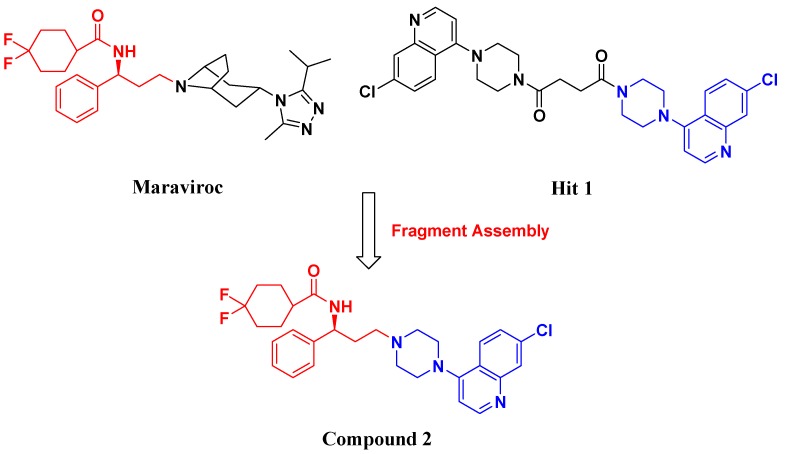
Design of novel scaffold **2** through fragment assembly.

In the last decade, numerous small molecule CCR5 antagonists have been reported. The discovery and development of CCR5 antagonists have been systmatically reviewed by Palani [13]. They include anilide-, oximino-piperidino-piperidine-, chiral piperazine-, tropane-, spirodiketopiperazine-, acyclic and cyclic scaffold-based compounds. These efforts have resulted recently in the FDA approval of the first small molecule CCR5 antagonist, maraviroc (Selzentry^®^, Figure 1) [14] for the treatment of HIV- 1 infection. But there are still various challenges and unknowns associated with CCR5 antagonists such as drug resistance, viral tropism and possible long term adverse events, so development of second generation CCR5 antagonists with improved properties is still much needed.

The fragment assembly method is a simply, effective structural modification strategy applied in medicinal chemistry field. These fragments are usually from active compounds or launched drugs, and possess comparatively good properties, such as high affinity, excellent metabolic stability, and low toxicity. Our CCR5 program was initiated from compound **1** (Figure 1) featuring the symmetric bis-4-(piperazin-1-yl)quinoline framework, and identified using a docking based virtual screening approach in conjunction with a calcium mobilization assay (IC_50_ = 2.00 μM). Mono-4-(piperazin-1-yl)quinoline derivatives have previously been reported as CCR5 antagonists [15]. By combining the attractive features of maraviroc and the 4-(piperazin-1-yl)quinoline moiety from hit **1**, and with the goal of identifying novel compounds that could be optimized to be more potent than compound **1**, compound **2** was identified via a calcium mobilization assay as a modest starting point (IC_50_ = 692 nM) for further optimization. Twelve derivatives **2a**-**l** of compound **2** were designed and synthesized, and their antagonistic activities against CCR5 were tested.

## Results and Discussion

### Identification of Compound ***1*** by Virtual Screening and Calcium Mobilization Assay

Targeting the optimized 3D model of CCR5 [16] which was constructed based on the bovine rhodopsin crystal structure (PDB entry 1F88) [17], a total of 80,000 compounds [18] in the SPECS database (http://www.specs.net/) were subsequently docked and ranked according to the scoring functions of DOCK 4.0 [19], the top-2,000 molecules were selected and re-scored by the scoring function of AutoDock 3.0 [20]. The 150 molecules with the highest score were selected from the SPECS database. Finally, 95 commercially available molecules were purchased and submitted to biological evaluation. Applying a calcium mobilization assay, compound **1** was identified among the above 95 compounds as a CCR5 antagonist (IC_50_ = 2.00 μM), so compound **1** was designated as a hit for further structural optimization.

**Scheme 1a:**
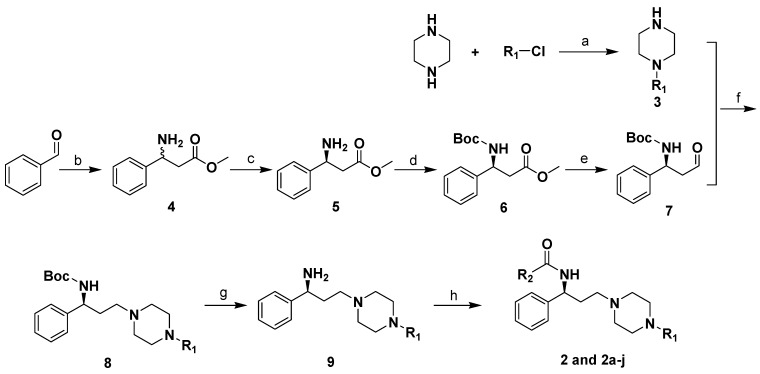
The synthetic route to compounds **2** and **2a**-**j**.

*^a^* *Reagents and conditions:* (a) K_2_CO_3_, DMF, N_2_, 45 °C; (b) (i) NH_4_OAc, EtOH, 45 °C, (ii) CH_2_(COOH)_2_, 60 °C, (iii) MeOH, SOCl_2_, 25 °C; (c) L(+)-tartaric acid, -20 °C; (d) (BOC)_2_O, Na_2_CO_3_, THF/H_2_O, 25 °C; (e) DIBAl-H, DCM, -78 °C; (f) NaBH(OAc)_3_, HOAc, DCM, 25 °C; (g) Saturated HCl in MeOH, 65 °C; (h) R_2_COOH, EDCI, HOBt, DIPEA, DCM, 25 °C or R_2_COCl, pyridine, 25 ºC. R_1_ and R_2_ are as in Table 1.

### Design and Synthesis of Compounds ***2*** and ***2a**-**l***

To discover novel compounds that could be optimized to be more potent than compound **1**, we designed novel chemical scaffold **2** through fragment assembly (Figure 1). Based on the structure of **2**, first, compounds **2a**-**f** were designed to prove if the 4,4-difluorocyclohexanecarbonyl unit is essential for antagonistic activity. Next, we changed the 7-chloroquinolin-4-yl of **2** for different heterocyclic aryl moieties (compounds **2g**-**j**) to examine if the heterocyclic aryl moiety would affect antagonistic activity. Lastly, compounds **2k**-**l** were prepared to investigate the importance of the chiral center and the (1-phenyl)propylamine spacer of **2**.

**Scheme 2a:**
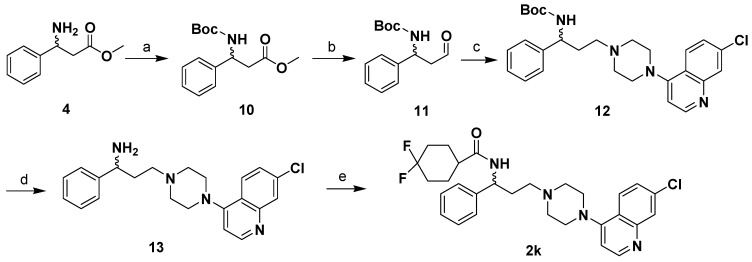
The synthetic route of compound **2k**.

*^a^* Reagents and conditions: (a) (BOC)_2_O, Na_2_CO_3_, THF/H_2_O, 25 °C; (b) DIBAL-H, DCM, -78 °C; (c) NaBH(OAc)_3_, HOAc, DCM, 25 °C; (d) Saturated HCl in MeOH, 65 °C; (e) 4,4- Difluorocyclohexanecarboxylic acid, EDCI, HOBt, DIPEA, DCM, 25 °C.

**Scheme 3a:**
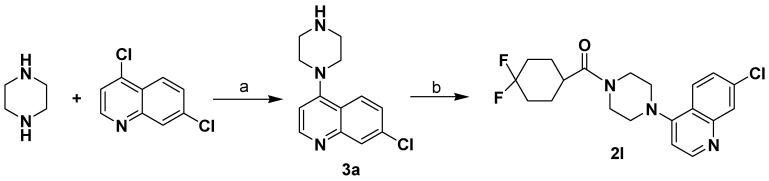
The synthetic route to compound **2l**.

*^a^* Reagents and conditions: (a) K_2_CO_3_, DMF, N_2_, 135 °C; (b) 4,4-Difluorocyclohexanecarboxylic acid, EDCI, HOBt, DIPEA, DCM, 25°C.

Compounds **2**, and **2a**-**l** were synthesized through the routes outlined in Scheme 1, Scheme 2 and Scheme 3, and the details for synthetic procedures and structural characterizations are described in the Experimental section.

### Calcium Mobilization Assay

For the primary assay, the percent inhibitory rates of the compounds **1**, **2** and **2a-l** at 1 μM were measured. Except compounds **2a** and **2c**, the other twelve compounds were remarkably antagonist of CCR5 activity. Therefore, we determined their IC_50_ values (Table 1). From the data in Table 1, we can see that antagonistic activity of compound **2** remarkably decreased 3-fold compared to that of compound **1** (IC_50_ from 2.00 μM down to 0.692 μM, respectively, Table 1).

**Table 1:** Chemical Structures of Compounds **1**, **2** and **2a-l** and Their Antagonistic Activities against CCR5.

Compound	Structure	IC_50_ (μM)
**1**	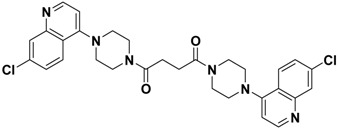	2.00
**2**	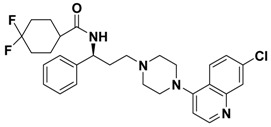	0.692
**2a**	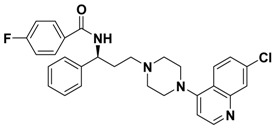	--
**2b**	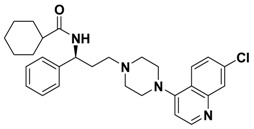	2.66
**2c**	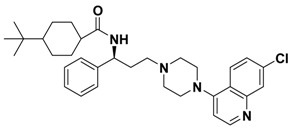	--
**2d**	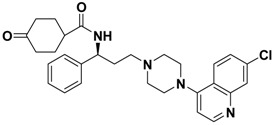	> 1.00
**2e**	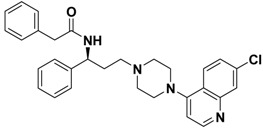	> 10.0
**2f**	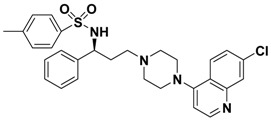	> 10.0
**2g**	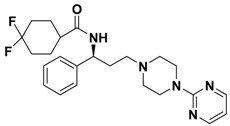	> 1.00
**2h**	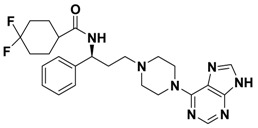	> 10.0
**2i**	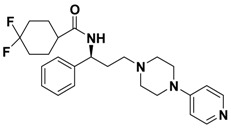	> 10.0
**2j**	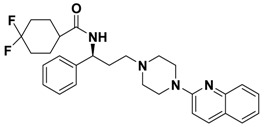	0.233
**2k**	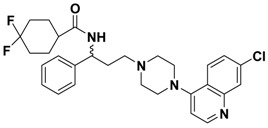	0.669
**2l**	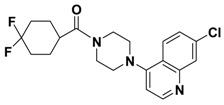	> 1.00
Maraviroc	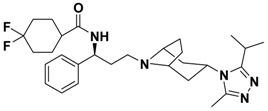	0.00261

### Structure and Activity Relationship Correction with the Binding Models

According to the above results, we can draw some SAR conclusions: (1) the 4,4-difluorocyclo-hexanecarbonyl of **2** (Table 1) is favorable for maintaining the antagonistic activity, as its replacement with various substituted carbonyls (compounds **2a**-**e**) or substituted sulfonyl (compound **2f**) resulted in decrease or loss of potency; (2) changing the 7-chloroquinolin-4-yl of **2** for some other heterocyclic aryl group (compounds **2g**-**j**) proved to be quite beneficial, resulting in a 3-fold increase in potency in the case of **2j** (IC_50_ from 0.692 μM down to 0.233 μM, respectively); (3) racemization of **2** (compound **2k**) has no effect on potency; (4) removing the (1-phenyl)propylamine spacer from **2** nearly leads to a loss of potency (compound **2l**). Although the best compound **2j** is 90-fold less potent than the control compound (maraviroc), these preliminary structure-activity relationships provide some valuable clues for structural optimization which could lead to development of useful second generation maraviroc- derived CCR5 antagonists.

**Figure 2:**
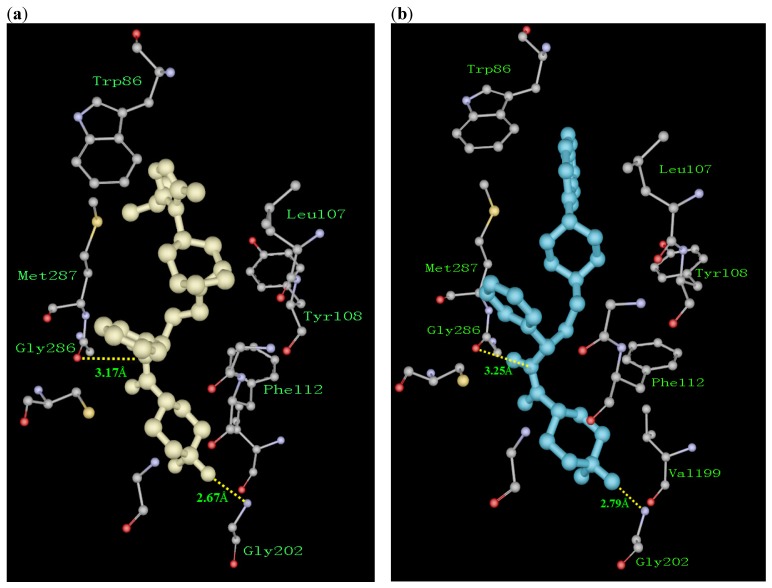
Detailed interactions of maraviroc (**a**) and **2j** (**b**) with the binding sites of CCR5. Compounds maraviroc and **2j** are indicated by yellow and blue thick sticks, respectively. Key residues of the binding pocket are shown as thin sticks. Hydrogen bonds are shown as *yellow dotted lines* with distance between donor and acceptor atoms. These pictures were prepared using ViewerPro (http://www.accelrys.com/).

To illustrate these SARs and gain structural information for further structural optimization, we compared the 3D binding models of the control compound maraviroc to CCR5 with that of the most potent antagonist **2j** to CCR5 generated based on the docking simulation (Figure 2a-b). Figure 2a shows that one fluorine of the cyclohexane ring and the amide N atom of maraviroc form two H-bond interactions with the Gly202 and Gly286 residues, respectively. Simultaneously, two excellent hydrophobic groups, the tropane moiety and the isopropyl group of maraviroc, form potent hydrophobic interactions with the Leu107, Tyr108, and Trp86 residues, respectively. Compound **2j** also forms two similar H-bond interactions with the Gly202 and Gly286 residues, whereas the piperazinyl group and quinoline ring of compound **2j** form weak hydrophobic interactions with the Leu107, Tyr108, and Trp86 residues, respectively. The above difference in strength of hydrophobic interactions between maraviroc and **2j** might partly affect the antagonistic activity. Considering that antagonistic activity of **2j** is ten times less potent than maraviroc, the definite interaction mechanism between antagonists and CCR5 needs further experimental verification. Inspired by molecular modeling, introduction some hydrophobic substituents to piperazinyl group and/or quinoline ring of compound **2j** would be likely to produce some additional hydrophobic interactions and be useful to improve activity.

## Conclusions

In this study, we have discovered a novel lead (**2j**) by using structure-based virtual screening approach in conjunction with chemical synthesis and bioassay. The preliminary SARs were obtained, which show the 4,4-difluorocyclohexanecarbonyl of compound **2** is necessary to maintain the antagonistic activity, and appropriate heterocyclic aryl of compound **2** could remarkably improve the antagonistic activity. These primary SARs are in good agreement with the molecular binding models and helpful for future antagonists design, and the novel scaffold presented here also provides potential application in development of maraviroc-derived second generation CCR5 antagonists.

## Experimental

### General

The chemicals used were purchased from Alfa, Acros and Shanghai Chemical Reagent Company, and used without further purification. Analytical thin-layer chromatography (TLC) was done on HSGF 254 plates (150-200 µm thickness, Yantai Huiyou Company, P.R. China). Yields were not optimized. Melting points were measured in capillary tubes on a SGW X-4 melting point apparatus without correction. Nuclear magnetic resonance (NMR) spectra were given on a Brucker AVANCE 500 NMR (TMS as IS). Chemical shifts were reported in parts per million (ppm, *δ*) downfield from tetramethylsilane. Proton coupling patterns were described as singlet (s), doublet (d), triplet (t), quartet (q), multiplet (m), and broad (br). Low- and high-resolution mass spectra (LRMS and HRMS) were given with electric ionization (EI) produced by a Finnigan MAT-95 instrument.

### Virtual Screening by Molecular Docking

The 3D molecular model was previously described [16]. Briefly, a homology model of the human CCR5 receptor was constructed based on the bovine rhodopsin crystal structure (PDB entry 1F88) [17] using the program Insight II (version 2000.1, Accelrys Inc., San Diego, CA, U.S.A.), and optimized using molecular mechanics method with the following parameters: a distance-dependent dielectric constant of 5.0; non-bonded cutoff of 8Å, Amber force field and Kollman all-atom charges [21]; and conjugate gradient minimization. The minimized structure was validated using the PROCHECK [22] program. The initial structures of the synthesized compounds (**2** and **2a**-**l**) and maraviroc were constructed with Insight II (version 2000.1, Accelrys Inc., San Diego, CA) and energetically minimized using Tripos force field with Gasteiger–Hückel charges [23]. The N-protonated forms of the molecules, which are the prevalent species at physiological pH, were used in the calculations.

The optimized 3D model of CCR5 was used as the target for virtual screening on database SPECS (http://www.specs.net/). The program DOCK4.0 [19] was employed for the primary screening. Residues around the catalytic center at radius of 6 Å was isolated for constructing the grids of docking screening, and the pocket composed by these residues was larger enough to include residues of the binding pocket. During the docking calculations, Kollman-all-atom charges [21] were assigned to the protein, and Geisterger-Hückel charges [23] were assigned to the small molecules in the SPECS databases. The conformational flexibility of the compounds from the database was considered in the docking searching.

The orientation of a ligand is evaluated with a shape scoring function and/or a function approximating the ligand-receptor binding energy. After the initial orientation and scoring evaluation, a grid-based rigid body minimization is carried out for the ligand to locate the nearest local energy minimum within the receptor binding site. The position and conformation of each docked molecule were optimized using single anchor search and torsion minimization method of DOCK4.0 [19].

Thirty configurations per ligand building a cycle and 50 maximum anchor orientations were used in the anchor-first docking algorithm. All docked configurations were energy minimized using 100 maximum iterations and 1 minimization cycle. Next, the top-2000 molecules were selected for further analyses. These molecules were re-scored by the scoring function of AutoDock3.0 [20]. Based on the second scoring results, 150 molecules were selected from the database. We purchased 95 available molecules for further bioassay.

### Calcium Mobilization Assay

CHO cells stably expressing CCR5 and Gα16 were loaded with 2 μmol/L Fluo-4 AM in Hanks balanced salt solution (HBSS, containing KCl 5.4 mM, Na_2_HPO_4_ 0.3 mM, KH_2_PO_4_ 0.4 mM, NaHCO_3_ 4.2 mM, CaCl_2_ 1.3 mM, MgCl_2_ 0.5 mM, Mg_2_SO_4_ 0.6 mM, NaCl 137 mM, BSA 5 g/L, Glucose 5.6 mM, Sulfinpyrazone 250 μM, pH 7.4) at 37 °C for 45 minutes. After the cells being rinsed with the reaction buffer, 50 μL HBSS containing known antagonists (positive control), compounds of interest or DMSO (negative control, final concentration 1%) were added. After incubation at room temperature for 10 minutes, 25 μL RANTES (final concentration 30 nM) was dispensed into the well using a FlexStation II micro-plate reader (Molecular Devices, Sunnyvale, CA, USA) and intracellular calcium change was recorded with an excitation wavelength of 485 nm and emission wavelength of 525 nm. The half maximal inhibitory concentrations (IC_50_s) of compounds were determined with GraphPad Prism software by constructing their dose-response curves.

*N-((S)-3-(4-(7-Chloroquinolin-4-yl)piperazin-1-yl)-1-phenylpropyl)-4,4-difluorocyclohexanecarbox-amide* (**2**). To a solution of (*S*)-3-(4-(7-chloroquinolin-4-yl)piperazin-1-yl)-1-phenylpropan-1-amine (**9**) (0.11 g, 0.29 mmol) and 4,4-difluorocyclohexanecarboxylic acid (0.06 g, 0.37 mmol) in DCM (5 mL) was added EDCI (0.07 g, 0.36 mmol), HOBt (0.05 g, 0.37 mmol) and DIPEA (0.15 mL, 0.85 mmol). The mixture was stirred for 4 h. The reaction mixture washed with brine and dried, then concentrated in vacuo. The residue was purified by flash column chromatography on silica gel, eluted with a mixture of MeOH/DCM (1:12, v/v), to afford **2** (0.12 g, 76%) as a yellow foam: mp 177-179 °C; ^1^H-NMR (CDCl_3_) *δ* 1.65-1.90 (m, 4H), 1.90-2.10 (m, 3H), 2.12-2.30 (m, 4H), 2.40-2.60 (m, 2H), 2.70-2.85 (m, 4H), 3.20-3.40 (m, 4H), 5.15 (q, 1H), 6.87 (d, 1H), 7.25-7.30 (m, 2H), 7.33-7.39 (m, 2H), 7.42-7.49 (m, 2H), 7.92 (d, 1H), 8.08 (d, 1H), 8.78 (d, 1H); EI-MS m/z 526 (M^+^), 260 (100%); HRMS (EI) m/z calcd. C_29_H_33_ClF_2_N_4_O (M^+^) 526.2311, found 526.2313.

*N-((S)-3-(4-(7-Chloroquinolin-4-yl)piperazin-1-yl)-1-phenylpropyl)-4-fluorobenzamide* (**2a**). Prepared in the same manner as described for **2** by replacing 4,4-difluorocyclohexanecarboxylic acid with 4- fluorobenzoic acid. Yellow foam: mp 77-79 °C; ^1^H-NMR(CDCl_3_) *δ* 2.12-2.30 (m, 2H), 2.50-2.62 (m, 2H), 2.72-2.90 (m, 4H), 3.15-3.30 (m, 4H), 5.38 (q, 1H), 6.81(d, 1H), 7.10 (t, 2H), 7.29 (m, 1H), 7.38 (m, 4H), 7.45 (dd, 1H), 7.90 (m, 3H), 8.08 (d, 1H), 8.78 (d, 1H); EI-MS 502 (M^+^), 274 (100%); HRMS (EI) m/z calcd. C_29_H_28_ClFN_4_O (M^+^) 502.1936, found 502.1932.

*N-((S)-3-(4-(7-Chloroquinolin-4-yl)piperazin-1-yl)-1-phenylpropyl)cyclohexanecarboxamide* (**2b**). Prepared as described for **2** using cyclohexanecarboxylic acid. Yellow solid: mp 153-158 °C; ^1^H- NMR (CDCl_3_,) *δ* 1.20-1.35 (m, 4H), 1.40-1.50 (m, 2H), 1.63-1.72 (m, 4H), 1.85-2.04 (m, 3H), 2.41-2.58 (m, 2H), 2.71-2.87 (m, 4H), 3.20-3.39 (m, 4H), 5.15 (q, 1H), 6.85 (d, 1H), 7.18-7.38 (m, 5H), 7.42 (dd, 1H), 7.91 (d, 1H), 8.06 (d, 1H), 8.72 (d, 1H); EI-MS 490 (M^+^), 274 (100%); HRMS (EI) m/z calcd. C_29_H_35_ClN_4_O (M^+^) 490.2499, found 490.2500.

*N-((S)-3-(4-(7-Chloroquinolin-4-yl)piperazin-1-yl)-1-henylpropyl)-4-tert-butylcyclohexanecarbox-amide* (**2c**). Prepared as described for **2** using 4-*tert*-butylcyclohexanecarboxylic acid. Yellow solid: mp 79-82 °C; ^1^H-NMR (CDCl_3_) *δ* 0.78 (9H, d), 1.00 (m, 2H), 1.21 (m, 1H), 1.45-1.65 (m, 4H), 1.80-2.00 (m, 2H), 2.10 (m, 1H), 2.15-2.25 (m, 2H), 2.45-2.65 (m, 2H), 2.75-2.90 (m, 4H), 3.28-3.40 (m, 4H), 5.15 (q, 1H), 6.87 (d, 1H), 7.23-7.38 (m, 5H), 7.47 (dd, 1H), 7.92 (d, 1H), 8.07 (d, 1H), 8.75 (d, 1H); EI-MS 546 (M^+^), 274 (100%); HRMS (EI) m/z calcd. C_33_H_43_ClN_4_O (M^+^) 546.3125, found 546.3127.

*N-((S)-3-(4-(7-Chloroquinolin-4-yl)piperazin-1-yl)-1-phenylpropyl)-4-oxocyclohexanecarboxamide* (**2d**). Prepared as described for **2** by replacing the 4,4-difluorocyclohexanecarboxylic acid with 4-oxo-cyclohexanecarboxylic acid. Yellow solid: mp 56-59 °C; ^1^H-NMR (CDCl_3_) *δ* 1.90-2.22 (m, 7H), 2.25- 2.35 (m, 2H), 2.40-2.60 (m, 4H), 2.75-2.90 (m, 4H), 3.20-3.32 (m, 4H), 5.12 (q, 1H), 6.87 (d, 1H), 7.28-7.40 (m, 5H), 7.45 (dd, 2H), 7.89 (d, 1H), 8.06 (d, 1H), 8.75 (d, 1H); EI-MS 504 (M^+^), 260 (100%); HRMS (EI) m/z calcd. C_29_H_33_ClN_4_O_2_ (M^+^) 504.2292, found 504.2293.

*N-((S)-3-(4-(7-Chloroquinolin-4-yl)piperazin-1-yl)-1-phenylpropyl)-2-phenylacetamide* (**2e**). To a solution of (S)-3-(4-(7-chloroquinolin-4-yl)piperazin-1-yl)-1-phenylpropan-1-amine (**9**, 0.09 g, 0.24 mmol) in DCM (5 mL) was added 2-phenylacetyl chloride (0.04 g, 0.26 mmol) and pyridine (2 drops), respectively. The mixture was stirred overnight at 25 °C and concentrated *in vacuo*. The residue was purified by flash column chromatography on silica gel, eluted with a mixture of MeOH/DCM (1:12, v/v), to afford **2e** (0.11 g, 92%) as a brown solid: mp 69-72 °C; ^1^H-NMR (CDCl_3_) *δ* 1.80-1.94 (m, 2H), 2.35-2.55 (m, 2H), 2.91-3.17 (m, 4H), 3.55-3.65 (m, 6H), 5.05 (q, 1H), 6.94 (d, 1H), 7.22-7.35 (m, 8H), 7.38-7.43 (m, 2H), 7.50 (dd, 1H), 7.79 (d, 1H), 8.18 (d, 1H), 8.75 (d, 1H); EI-MS 498 (M^+^), 274 (100%); HRMS (EI) m/z calcd. C_30_H_31_ClN_4_O (M^+^) 498.2186, found 498.2183.

*N-((S)-3-(4-(7-Chloroquinolin-4-yl)piperazin-1-yl)-1-phenylpropyl)-4-methylbenzenesulfonamide* (**2f**). Prepared in the same manner as described for **2e** by replacing the 2-phenylacetyl chloride with *p*-toluenesulfonyl chloride. Yellow solid: mp 132-136 °C; ^1^H-NMR (CDCl_3_) *δ* 2.08-2.15 (m, 2H), 2.32 (s, 3H), 2.80-3.10 (m, 6H), 3.51-3.63 (m, 4H), 4.53 (q, 1H), 6.90 (d, 1H), 7.05-7.19 (m, 7H), 7.45-7.55 (m, 3H), 7.88 (d, 1H), 8.18 (d, 1H), 8.74 (d, 1H); EI-MS 534 (M^+^), 260 (100%); HRMS (EI) m/z calcd. C_29_H_31_ClN_4_O_2_S (M^+^) 534.1856, found 534.1858.

*N-((S)-3-(4-(Pyrimidin-2-yl)piperazin-1-yl)-1-phenylpropyl)-4,4-difluorocyclohexanecarboxamide* (**2g**). Replacing 4,7-dichloroquinoline with 4-chloropyrimidine, in the same manner as described for **2**, **2g** was prepared as a yellow oil; ^1^H-NMR(CDCl_3_) *δ* 1.69-2.10 (m, 7H), 2.10-2.30 (m, 4H), 2.40-2.50 (m, 2H), 2.60-2.75 (m, 4H), 3.80-3.97 (m, 4H), 5.10 (q, 1H), 6.51 (t, 1H), 7.25-7.35 (m, 5H), 8.31 (d, 2H); EI-MS 443 (M^+^), 177 (100%); HRMS (EI) m/z calcd. C_24_H_31_F_2_N_5_O (M^+^) 443.2497, found 443.2498.

*N-((S)-3-(4-(9H-Purin-6-yl)piperazin-1-yl)-1-phenylpropyl)-4,4-difluorocyclohexanecarboxamide* (**2h**). Replacing 4,7-dichloroquinoline with 6-chloro-9*H*-purine, in the same manner as described for **2**, **2h** was prepared as a white soild; ^1^H-NMR (CDCl_3_) *δ* 1.70-2.20 (m, 11H), 2.40-2.52 (m, 2H), 2.70-2.85 (m, 4H), 4.30-4.50 (m, 4H), 5.10 (q, 1H), 7.25-7.37 (m, 5H), 7.91 (s, 1H), 8.34 (s, 1H); EI-MS 483 (M^+^), 231 (100%); HRMS (EI) m/z calcd. C_25_H_31_F_2_N_7_O (M^+^) 483.2558, found 483.2559.

*N-((S)-3-(4-(pyridin-4-yl)piperazin-1-yl)-1-phenylpropyl)-4,4-difluorocyclohexanecarboxamide* (**2i**). Replacing 4,7-dichloroquinoline with 4-chloropyridine, in the same manner as described for **2**, **2i** was prepared as a brown oil; ^1^H-NMR (CDCl_3_) *δ* 1.65-2.20 (m, 9H), 2.25-2.36 (m, 4H), 2.40-2.40 (m, 4H), 3.23-3.42 (m, 4H), 5.12 (q, 1H), 6.87 (d, 2H), 7.24-7.30 (m, 2H), 7.38-7.43 (m, 3H), 8.78 (d, 2H); EI-MS 442 (M^+^), 106 (100%); HRMS (EI) m/z calcd. C_25_H_32_F_2_N_4_O (M^+^) 442.2544, found 442.2540.

*N-((S)-3-(4-(Quinolin-2-yl)piperazin-1-yl)-1-phenylpropyl)-4,4-difluorocyclohexanecarboxamide* (**2j**). Replacing 4,7-dichloroquinoline with 2-chloroquinoline, in the same manner as described for **2**, **2j** was prepared as a brown soild: mp 50-53 °C; ^1^H-NMR (CDCl_3_) *δ* 1.65-2.40 (m, 11H), 2.45-2.63 (m, 2H), 2.70-2.94 (m, 4H), 3.80-3.94 (m, 4H), 5.08 (q, 1H), 6.98 (d, 1H), 7.25-7.38 (m, 6H), 7.58 (t, 1H), 7.63 (d, 1H), 7.73 (d, 1H), 7.95 (d, 1H); EI-MS 492 (M^+^), 157 (100%); HRMS (EI) m/z calcd. C_29_H_34_F_2_N_4_O (M^+^) 492.2701, found 492.2700.

*N-(3-(4-(7-Chloroquinolin-4-yl)piperazin-1-yl)-1-phenylpropyl)-4,4-difluorocyclohexanecarboxamide* (**2k**). Replacing (*S*)-methyl 3-amino-3-phenylpropanoate (**5**) with (±)-methyl 3-amino-3- phenylpropanoate (**4**), in the same manner as described for **2**, **2k** was prepared as a brown solid: mp 177-120 °C; ^1^H-NMR (CDCl_3_) *δ* 1.67-1.89 (m, 4H), 1.90-2.33 (m, 7H), 2.59-2.65 (m, 2H), 2.80-2.95 (m, 4H), 3.30-3.40 (m, 4H), 5.15 (q, 1H), 6.88 (d, 1H), 7.25-7.30 (m, 2H), 7.33-7.39 (m, 3H), 7.46 (dd, 1H), 7.92 (d, 1H), 8.08 (d, 1H), 8.78 (d, 1H); EI-MS 526 (M^+^), 260 (100%); HRMS (EI) m/z calcd. C_29_H_33_ClF_2_N_4_O (M^+^) 526.2311, found 526.2310.

*(4-(7-chloroquinolin-4-yl)piperazin-1-yl)(4,4-difluorocyclohexyl)methanone* (**2l**). Replacing (*S*)-3-(4- (7-chloroquinolin-4-yl)piperazin-1-yl)-1-phenylpropan-1-amine (**9**) with 7-chloro-4-(piperazin-1- yl)quinoline (**3a**), in the same manner as described for **2**, **2l** was prepared as a yellow oil; ^1^H- NMR (CDCl_3_,) *δ* 1.65-2.02 (m, 5H), 2.16-2.32 (m, 4H), 3.16-3.30 (m, 4H), 3.70-3.90 (m, 4H), 6.87 (d, 1H), 7.50 (dd, 1H), 7.98 (d, 1H), 8.12 (d, 1H), 8.76 (d, 1H); EI-MS 393 (M^+^), 217 (100%); HRMS (EI) m/z calcd. C_20_H_22_ClF_2_N_3_O (M^+^) 393.1419, found 393.1417.

*7-Chloro-4-(piperazin-1-yl)quinoline* (**3a**). A mixture of 4,7-dichloroquinoline (0.5 g, 2.5 mmol), piperazine (2.5 g, 12.6 mmol), K_2_CO_3_ (0.1 g, 0.72 mmol), triethylamine (0.1 mL, 0.71 mmol) in *N*-methyl-2-pyrrolidinone (10 mL) was stirred under nitrogen at 135 °C for 4 h. After cooling to room temperature, the mixture was diluted with CH_2_Cl_2_ (20mL) and washed with brine. The organic layer was separated, dried, filtered and condensed. The residue was purified by flash column chromatography on silica gel, eluted with a mixture of MeOH/EtOAc (1:4, v/v), to afford **3a** (0.33 g, 54%) as a white solid: mp 113-114 °C ; ^1^H-NMR (CDCl_3_): *δ* 3.08 (m, 8H), 6.85 (d, 1H), 7.40 (dd, 1H), 7.91 (d, 1H), 8.09 (d, 1H), 8.70 (d, 1H).

*(±)-Methyl 3-amino-3-phenylpropanoate* (**4**). Benzaldehyde (5 g, 47.2 mmol) and NH_4_OAc (7.3 g, 94.8 mmol) were dissolved in EtOH (50 mL). The solution was stirred at 45 °C for 12 h and then was added to a solution of malonic acid (4.9 g, 47.1 mmol) in EtOH (25 mL). The mixture was stirred overnight at 60 °C and then at reflux for 6 h. The reaction mixture was cooled to 5 °C. The resulting precipitate was collected by filtration and washed with ice-cold EtOH. The white solid was dried in vacuo, to give 3-amino-3-phenylpropionic acid. The acid was dissolved in MeOH (25 mL) and cooled to 5 °C. To this mixture was added dropwise SOCl_2_ (2.5 mL) and the mixture was stirred overnight at r.t. The solution was refluxed for 2h and subsequently concentrated to dryness. To the white residue was added 2 M Na_2_CO_3_ (20 mL) and the mixture was extracted with EtOAc (3 x 20 mL). The combined organic layers were dried over Na_2_SO_4_ and concentrated in vacuo, to give **4** (3.88 g, 46%) as white oil. ^1^H-NMR (CDCl_3_) *δ* 1.82 (s, 2H), 2.70 (d, 2H), 3.70 (s, 3H), 4.45 (t, 1H), 7.28 (m, 1H), 7.38 (m, 4H).

*(S)-Methyl 3-amino-3-phenylpropanoate* (**5**). A solution of **4** (1 g, 5.6 mmol) in MeOH (5 mL) was added to a refluxing soln. of (+)-L-tartaric acid (0.84 g, 5.6 mmol) in MeOH (6 mL). The product crystallized overnight at -20 °C and was filtered, to give tartrate of **5** as a white powder, mp 174-176 °C (Lit. [24] 170.6-171.6 °C). The tartrate was dissolved in 1 N aq. NaOH, then was extracted with CHCl_3_ (3 x 10 mL). The organic layer was dried with Na_2_SO_4_ and concentrated, to give **5** (0.30 g, 60%) as a colorless oil. [α]^28^_D_ = -15.6 (c = 1.13 in CH_2_Cl_2_) (Lit. [24] [α]^22^_D_ = -20.3 (c = 1.53 in CHCl_3_)); ^1^HNMR (CDCl_3_) *δ* 2.85 (m, 1H), 2.95 (m, 1H), 3.68 (s, 3H), 4. 52 (dd, 1H), 7.16-7.48 (m, 5H).NMR (CDCl_3_) *δ* 2.85 (m, 1H), 2.95 (m, 1H), 3.68 (s, 3H), 4. 52 (dd, 1H), 7.16-7.48 (m, 5H).

*tert-Butyl (S)-2-(methoxycarbonyl)-1-phenylethylcarbamate* (**6**). To a cold (0 °C) mixture of Na_2_CO_3_ (2.8 g, 26.5 mmol) in THF/H_2_O (5 mL, 2:1, v/v) was added **5** in one portion (0.85 g, 4.7 mmol). A solution of (BOC)_2_O (1.2 g, 5.5 mmol) in THF (4 mL) was added in one portion and the reaction stirred at 0 °C for 1 h, then at 25 °C for 2 h, extracted with EtOAc (3 x 20 mL). The combined organic extracts were dried and concentrated *in vacuo*. The crude white solid product **6** was used in the next step without further purification.

*tert-Butyl (S)-2-formyl-1-phenylethylcarbamate* (**7**). To a soln. of **6** (1.5 g, 5.4 mmol) in DCM (10 mL) cooled to -78 °C was added DIBAL-H (8.7 mL of 25% W/W in hexane, 12.0 mmol) dropwise at a rate that maintained the temperature below -70 °C. After 2 h the reaction was quenched by the addition of MeOH (1.7 mL) and H_2_O (1 mL) at -78 °C, then allowed to warm to 25 °C. The mixture was filtered. The filtrate was dried and concentrated in vacuo. The crude colorless oil **7** (0.76 g, 56%) was used in the next step without further purification. ^1^H-NMR (CDCl_3_) *δ* 1.50 (s, 9H), 2.85-3.05 (m, 2H), 5.10 (s, 1H), 7.25-7.40 (m, 5H), 9.80 (s, 1H).

*Tert-butyl (S)-3-(4-(7-chloroquinolin-4-yl)piperazin-1-yl)-1-phenylpropylcarbamate* (**8**). To a solution of **7** (0.25 g, 1 mmol), **3a** (0.25 g, 1 mmol) and HOAc (0.14 mL) in DCM (10 mL) was added NaBH(OAc)_3_ (0.26 g, 1.2 mmol) in one portion and the mixture was stirred for 5 h. The reaction was quenched by adding 10% K_2_CO_3_ soln. (20 mL) and stirred for an additional 30 min, extracted with DCM (3 x 20 mL). The combined organic layer was dried and concentrated in vacuo. The residue was purified by flash column chromatography on silica gel, eluted with a mixture of MeOH/DCM (1:12, v/v), to afford **8** (0.32 g, 67%) as a yellow foam. ^1^H-NMR (CDCl_3_) *δ* 1.35 (s, 9H), 2.15 (m, 2H), 2.61 (m, 2H), 2.91 (m, 4H), 3.35 (s, 4H), 4.69 (m, 1H), 6.80 (d, 1H), 7.22-7.32 (m, 5H), 7.40 (dd, 1H), 7.81 (d, 1H), 8.08 (d, 1H), 8.65 (d, 1H).

*(S)-3-(4-(7-Chloroquinolin-4-yl)piperazin-1-yl)-1-phenylpropan-1-amine* (**9**). A solution of **8** (0.19 g, 0.40 mmol) in saturated HCl in MeOH (6 mL) heated at 65 °C for 2 h. The MeOH was evaporated under reduced pressure and the residue cautiously partitioned between DCM (20 mL) and 20% K_2_CO_3_ solution (20 mL). The aqueous layer was re-extracted with DCM (3 x 20mL). The combined organic layer was dried and concentrated in vacuo, to afford **9** (0.14 g, 93%) as a straw yellow oil which was used in the next step without further purification. ^1^H NMR (CDCl_3_) *δ* 1.98-2.12 (m, 2H), 2.58 (m, 2H), 2.81 (m, 4H), 3.25 (m, 4H), 4.18 (m, 1H), 6.81 (d, 1H), 7.20 (t, 1H), 7.32 (t, 2H), 7.35-7.44 (m, 3H), 7.90 (d, 1H), 8.05 (d, 1H), 8.68 (d, 1H).
